# Supplementary material for: A UMLS-based spell checker for natural language processing in vaccine safety
Source: BMC Med Inform Decis Mak. 2007 Feb 12;7:3. doi: 10.1186/1472-6947-7-3 (PMC1805499; doi:10.1186/1472-6947-7-3)
Supplement: Additional file 13 — RAP application directory. Sets up directory for RAP (RDF application for PHP) source code [file 1472-6947-7-3-S13.gz › rap/doc/phpdoc/__filesource/fsource_utility__utilStatementIterator.php.html]

xml version="1.0" encoding="iso-8859-1"?


File Source for StatementIterator.php


# Source for file StatementIterator.php

Documentation is available at StatementIterator.php

```
1. <?php

3. // ----------------------------------------------------------------------------------
4. // Class: StatementIterator
5. // ----------------------------------------------------------------------------------


9. /**
10. * Iterator for traversing models.
11. * This class can be used for iterating forward and backward trough MemModels.
12. * It should be instanced using the getIterator() method of a MemModel.
13. *
14. * <BR><BR>History:<UL>
15. * <LI>08-10-2004                 : Class completely rewritten</LI>
16. * <LI>03-29-2004                 : Problems with adding and removing statements fixed.</LI>
17. * <LI>02-21-2003                : First version of this class.</LI>
18. * </UL>
19. *
20. * @version  V0.9.1
21. * @author Daniel Westphal <mail at d-westphal dot de>
22. * @author Chris Bizer <chris@bizer.de>
23. *
24. * @package utility
25. * @access    public
26. *
27. */
28. class StatementIterator extends Object {

30. /**
31. * Reference to the MemModel
32. * @var        object MemModel
33. * @access    private
34. */
35. var $model;

37. /**
38. * Current position
39. * StatementIterator does not use the build in PHP array iterator,
40. * so you can use serveral iterators on a single MemModel.
41. * @var        integer
42. * @access    private
43. */
44. var $position;


47. /**
48. * Constructor
49. *
50. * @param    object    MemModel
51. * @access    public
52. */
53. function StatementIterator(&$model) {
54. $this->model = $model;
55. reset($this->model->triples);
56. $this->position = -1;
57. }

59. /**
60. * Returns TRUE if there are more statements.
61. * @return    boolean
62. * @access    public
63. */
64. function hasNext() {
65. if ($this->position == -1)
66. {
67. if(current($this->model->triples)!=NULL)
68. {
69. return TRUE;
70. } else
71. {
72. return FALSE;
73. }
74. };

76. if (next($this->model->triples)!=NULL )
77. {
78. prev($this->model->triples);
79. return TRUE;
80. } else
81. {
82. end($this->model->triples);
83. return FALSE;
84. };
85. }

87. /**
88. * Returns TRUE if the first statement has not been reached.
89. * @return    boolean
90. * @access    public
91. */
92. function hasPrevious() {
93. if (prev($this->model->triples)!=NULL)
94. {
95. next($this->model->triples);
96. return TRUE;
97. } else {
98. reset($this->model->triples);
99. return FALSE;
100. };
101. }

103. /**
104. * Returns the next statement.
105. * @return    statement or NULL if there is no next statement.
106. * @access    public
107. */
108. function next() {
109. if ($this->position == -1)
110. {
111. if($return=current($this->model->triples))
112. {
113. $this->position=key($this->model->triples);
114. return $return;
115. } else
116. {
117. $this->position=key($this->model->triples);
118. return NULL;
119. };
120. };


123. if ($return=next($this->model->triples)) {
124. $this->position=key($this->model->triples);
125. return $return;
126. } else {
127. $this->position=key($this->model->triples);
128. return NULL;
129. };
130. }

132. /**
133. * Returns the previous statement.
134. * @return    statement or NULL if there is no previous statement.
135. * @access    public
136. */
137. function previous() {
138. if ($return=prev($this->model->triples)) {
139. $this->position=key($this->model->triples);
140. return $return;
141. } else {
142. $this->position=key($this->model->triples);
143. return NULL;
144. }
145. }

147. /**
148. * Returns the current statement.
149. * @return    statement or NULL if there is no current statement.
150. * @access    public
151. */
152. function current() {
153. if ($this->position==-1) return NULL;

155. if ($return=current($this->model->triples)) {
156. return $return;
157. } else {
158. return NULL;
159. }
160. }

162. /**
163. * Moves the pointer to the first statement.
164. * @return    void
165. * @access    public
166. */
167. function moveFirst() {
168. reset($this->model->triples);
169. $this->position=0;
170. }

172. /**
173. * Moves the pointer to the last statement.
174. * @return    void
175. * @access    public
176. */
177. function moveLast() {
178. end($this->model->triples);
179. $this->position=key($this->model->triples);
180. }

182. /**
183. * Moves the pointer to a specific statement.
184. * If you set an off-bounds value, the position will be set to the last element
185. * @param     integer
186. * @return    void
187. * @access    public
188. */
189. function moveTo($position) {

191. if ($position + 1 > count($this->model->triples))
192. {
193. end($this->model->triples);
194. } else
195. {
196. reset($this->model->triples);

198. for ($i = 1; $i < $position; $i++) {
199. next($this->model->triples);
200. }
201. };
202. $this->position=key($this->model->triples);
203. }

205. /**
206. * Returns the current position of the iterator.
207. * @return    integer
208. * @access    public
209. */
210. function getCurrentPosition() {
211. return key($this->model->triples);
212. }

214. }

216. ?>
```

Documentation generated on Thu, 7 Jul 2005 13:43:58 +0200 by phpDocumentor 1.3.0RC3
